# Best practices for biotin ligase–based proximity labeling proteomics in plant systems

**DOI:** 10.1093/plcell/koag257

**Published:** 2026-09-01

**Authors:** Yiling Fang, Jacob Moe-Lange, Shou-Ling Xu, Nitzan Shabek, Yangnan Gu

**Affiliations:** Department of Plant and Microbial Biology, University of California, 371 Koshland Hall, Berkeley, CA 94720, United States; Department of Plant Biology, College of Biological Sciences, University of California, 1224 Green Hall, Davis, CA 95616, United States; Department of Biology and Carnegie Mass Spectrometry Facility, Carnegie Institution for Science, 260 Panama Street, Stanford, CA 94305, United States; Department of Plant Biology, College of Biological Sciences, University of California, 1224 Green Hall, Davis, CA 95616, United States; Department of Plant and Microbial Biology, University of California, 371 Koshland Hall, Berkeley, CA 94720, United States

## Abstract

Proximity labeling (PL) proteomics, primarily powered by engineered biotin ligases such as BioID and TurboID, has emerged as a transformative approach for mapping protein association networks and subcellular proteomes in living cells. By covalently biotinylating proteins within a nanometer-scale radius of a bait protein, PL captures transient, weak, and spatially restricted associations that often escape conventional affinity-based methods. However, applying PL in plants introduces distinctive challenges, including rigid cell walls that can limit biotin penetration, tissue-specific variation in substrate accessibility, enzyme temperature sensitivity, and the difficulty of removing excess free biotin after labeling. Here, we outline best practices for designing and implementing biotin ligase-based PL experiments in plant systems, drawing on experience across multiple species. We discuss key considerations, including enzyme selection, expression system design, fusion protein validation, biotin delivery strategies, protein extraction and enrichment, and mass spectrometry–based analysis. We also highlight emerging quantitative and conditional PL approaches that enable dynamic comparison of proxiomes across developmental stages, environmental conditions, and genetic backgrounds. Throughout, we emphasize the importance of rigorous controls, careful terminology, and orthogonal validation to ensure biologically meaningful interpretation of PL datasets. These guidelines aim to standardize experimental design and interpretation, facilitating reproducible and biologically meaningful PL studies in plant systems.

## Introduction

A comprehensive understanding of cellular biology requires the systematic characterization of molecular components across defined subcellular locations and time points. Protein–protein interactions (PPIs) underpin virtually all cellular processes and frequently provide critical functional insight into both poorly characterized and well-studied proteins, revealing unexpected regulatory modules, pathway crosstalk, and context-dependent functions. These opportunities continue to motivate the development of diverse experimental approaches to interrogate PPIs.

Affinity-based methods, such as immunoprecipitation coupled with mass spectrometry (IP-MS) or co-fractionation coupled with mass spectrometry (CF-MS), have proven instrumental in delineating PPI networks; however, both generally require robust (often ectopic) protein expression and favor the detection of stable complexes. As a result, transient, weak, or spatially restricted interactions are frequently missed, particularly those occurring within membrane-bound organelles and structures or highly dynamic subcellular compartments such as biomolecular condensates. Targeted approaches like bimolecular fluorescence complementation (BiFC) can visualize PPI in living cells and partially address issues mentioned above; however, they are not suited for unbiased, proteome-scale interactor discovery (Kerppola 2006; Kudla and Bock 2016).

Proximity labeling (PL) technologies have substantially expanded the experimental toolkit for studying protein function in vivo (Mair and Bergmann 2022; Pfeiffer et al. 2022; Xu et al. 2023; Milione et al. 2024; Sun et al. 2024; Hamada et al. 2026). PL approaches exploit engineered enzymes, most notably biotin ligases (eg, BioID2, TurboID, miniTurbo) that catalyze the covalent biotinylation of proteins within a defined nanometer-scale radius (typically 10 to 20 nm) of a bait protein (Sears et al. 2019). Because labeling occurs in situ under near-physiological conditions, and enrichment of labeled proteins does not require the preservation of intact protein complexes, PL is well suited for capturing transient associations and weak binding partners in their native cellular context. These same properties make PL particularly powerful for profiling the proteomes of subcellular compartments that are notoriously refractory to traditional biochemical purification, such as mitochondrial intermembrane space, the nuclear envelope, and the diverse array of membraneless biomolecular condensates (Huang et al. 2020; Tang et al. 2020; Jia et al. 2023; Liu et al. 2023; Dragwidge et al. 2024; Tan et al. 2024; Tang et al. 2024; Kenneally et al. 2026).

A defining strength of PL is its inherent versatility, allowing researchers to tailor the labeling chemistry to specific biological questions. Historically, a key advantage of PL has been its capacity for signal accumulation during the labeling period. Because the enzyme continuously biotinylates proteins in the bait's vicinity, a single bait molecule can modify multiple neighbors, while individual substrates can acquire multiple biotin moieties. This cumulative approach maximizes detection sensitivity and captures a comprehensive protein “interaction history,” including transient, weak, sequential, or mutually exclusive contacts that collectively define the broader local proteomic environment (Qin et al. 2021). Conversely, there is a growing emphasis on optimizing PL for high spatiotemporal resolution. By leveraging more kinetically active enzyme or emerging chemical labeling strategies, such as photocatalyst-driven carbene generation, together with tightly restricted labeling windows, researchers can generate precise snapshots of specific signaling events or microenvironments while minimizing the capture of bystander proteins in the crowded cellular milieu (Geri et al. 2020; Bartholow et al. 2024). Rather than being mutually exclusive, these approaches occupy a methodological spectrum: long-term accumulation provides a robust and comprehensive map of the local cellular neighborhood, whereas short-burst labeling affords higher spatial and temporal resolution.

Despite the considerable advantages for mapping protein composition and interaction networks under diverse physiological and developmental conditions, the application of PL in plants introduces unique experimental challenges. These include rigid cell walls that complicate efficient lysis and biotin penetration, as well as tissue-specific variation in biotin substrate accessibility. In addition, plant tissues are often sensitive to elevated temperatures, which correspond to the optimal operating temperatures of some biotin ligases. Some plant species also contain high levels of endogenous biotin, increasing the risk of auto-labeling, and plant systems can present difficulties in efficiently removing free biotin after labeling. Although PL has been successfully deployed across diverse plant model systems, including *Arabidopsis thaliana* (Mair et al. 2019; Tang and Gu, 2025), *Oryza sativa* (Lin et al. 2017; Shi et al. 2024; Zhang et al. 2026), *Nicotiana benthamiana* (Mair et al. 2019; Zhang et al. 2019; Zhang et al. 2023; Hamada et al. 2026), *Brassica oleracea* (Zhang et al. 2026), and *Chlamydomonas reinhardtii* (Kreis et al. 2023; Lau et al. 2023; Tulin et al. 2025), each system demands careful optimization of enzyme selection, labeling conditions, and enrichment protocols, together with rigorous experimental controls and cautious data interpretation. Here, we synthesize emerging community best practices spanning experimental design, execution, data analysis, and reporting to provide a practical and reproducible framework for the biologically meaningful application of biotin ligase-based PL technologies in plant biology.

## The expanding toolkit of proximity labeling technologies

### The development of biotin ligase-based proximity labeling tools

The first biotin-based PL method, BioID, was introduced in 2012 using a promiscuous mutant of the *Escherichia coli* biotin ligase BirA (BirA*, harboring an R118G substitution) that allows activated biotinoyl-5′-AMP to dissociate and covalently label lysine residues on neighboring proteins within ∼10 nm (Roux et al. 2012). Cells that express a fusion of the bait protein and BirA* are provided with exogenous biotin. Over a period of 16 to 24 h, proximal proteins undergo biotinylation, and these labeled proteins are subsequently isolated through streptavidin affinity purification for analysis via MS. BioID2, derived from *Aquifex aeolicus* biotin ligase, offers a smaller footprint (27 kDa vs. 35 kDa) and improved labeling efficiency at lower biotin concentrations (Kim et al. 2016). Both variants have been widely applied in plants to map protein interactomes and complex composition (Conlan et al. 2018; Khan et al. 2018; Huang et al. 2020; Tang et al. 2020, 2022b; Miltenburg et al. 2022; Jia et al. 2023).

A key limitation of BirA* and BioID2 for plant applications is their slow labeling kinetics and temperature sensitivity—peak activity occurs at 37 °C, well above typical growth conditions for model plants such as Arabidopsis (22 °C). To overcome this, directed evolution via yeast display produced TurboID and miniTurbo (Branon et al. 2018). TurboID (carrying 15 mutations relative to wild-type BirA) achieves robust biotinylation within minutes across a range of physiological temperatures and has been validated in multiple plant systems and green algae (Mair et al. 2019; Zhang et al. 2019; Xu et al. 2021; Tang et al. 2022a; Kreis et al. 2023; Fang et al. 2025; Rui et al. 2026). miniTurbo (N-terminal deletion of TurboID with 13 mutations) produces lower background labeling in the absence of exogenous biotin, affording better temporal control, though at the cost of reduced overall labeling signal (Mair et al. 2019; Tang et al. 2024).

More recently, additional variants have been developed to balance activity, specificity, and tag size. AirID, reconstructed de novo from ancestral BirA sequences, achieves stronger labeling than BirA* in 3 h while exhibiting less nonspecific background than TurboID (Kido et al. 2020); its feasibility in plants has been demonstrated via agroinfiltration in cucumber (Zada et al. 2022). UltraID, an evolved fragment of BioID2, matches TurboID in biotinylation efficiency while substantially reducing tag size, an important consideration for membrane-associated or structurally constrained interactomes where large fusion tags may perturb protein function or complex assembly (Kubitz et al. 2022). Although neither AirID nor UltraID has yet seen broad adoption in plant systems, both represent promising options, particularly when tag size or background labeling is a concern.

### Advanced applications of biotin ligase-based proximity labeling

Beyond conventional bait-centric PL, several specialized strategies have been developed to interrogate specific interaction contexts with greater precision. Split-BioID divides the BirA* enzyme into 2 complementary fragments, each fused to one protein of interest; enzymatic activity is reconstituted only upon interaction between the 2 bait proteins, enabling selective biotinylation of proteins associated with a defined complex rather than either bait alone (De Munter et al. 2017; Schopp et al. 2017; Kwak et al. 2020). Split-TurboID was subsequently developed, combining the conditional reconstitution strategy with the rapid labeling kinetics of TurboID (Cho et al. 2020a; Cho et al. 2020b). Split-TurboID has been successfully applied to map proteomes at endoplasmic reticulum–mitochondria contact sites in mammalian cell lines, characterize basal cell polarity modules in Arabidopsis, and profile protein complexes formed pre- and post-immune activation in *N. benthamiana*, demonstrating its utility for studying spatially restricted and temporally regulated protein assemblies (Cho et al. 2020b; Zhang et al. 2024; Huang et al. 2025)

For chromatin-associated protein discovery, CRISPR-based PL leverages nuclease-dead Cas9 (dCas9) to direct TurboID to user-defined genomic loci via single-guide RNAs. This approach enables unbiased identification of transcription factors, chromatin remodelers, and other regulatory factors enriched at specific DNA sequences and complements existing methods such as ChIP-seq and CUT&RUN, which require prior knowledge of the DNA-binding protein and may not reveal the full complement of locus-associated factors (Park 2009; Skene and Henikoff 2017; Cenik et al. 2024). Encouragingly, CRISPR-based PL has recently been implemented in plant systems, including *A. thaliana*, *N. benthamiana*, *B. oleracea*, and *O. sativa* (Zhang et al., 2025a; Zhang et al. 2026). Despite its promise, several limitations should be considered when interpreting CRISPR-based PL datasets. In particular, off-target binding of dCas9 may recruit the labeling enzyme to unintended genomic regions, potentially resulting in the identification of proteins unrelated to the target locus. In addition, the spatial labeling radius of TurboID can encompass proteins associated with neighboring chromatin regions, limiting the ability to distinguish factors directly bound to the target sequence from those located nearby in three-dimensional chromatin space. Careful guide RNA design, appropriate controls, and orthogonal validation experiments are therefore essential to establish locus-specific protein associations.

PL has also been extended to RNA–protein interactions through RapID, which utilizes a biotin ligase fused to the λN peptide that can recognize the BoxB stem-loop structures flanking the RNA sequence of interest (Ramanathan et al. 2018). RapID employs BASU, a *Bacillus subtilis*-derived BirA* variant, which offers improved labeling speed for RapID. BASU generated a strong biotinylation signal within 1 min of biotin treatment in HEK293T cells. Although RapID has not yet been applied in plant systems, it offers considerable potential for dissecting RNA–protein regulatory networks, identifying sequence-specific RNA-binding factors, and uncovering host proteins that associate with viral RNAs.

## Principles of experimental design

### Selecting the appropriate enzyme

PL is most informative when the experimental objective is clearly defined—whether to map a protein's physical proxiome, characterize the proteome of a subcellular compartment, or capture dynamic interaction partners in response to developmental or environmental stimuli. The choice of labeling enzyme should follow directly from this objective, as different biotin ligases vary substantially in catalytic rate, temperature optimum, size, and background labeling, all of which carry practical consequences in plant systems (Table 1).

**Table 1:** Summary of biotin ligase enzymes and experimental protocols in plants.

Enzyme	Size	Species	Temperature	Tissue	Biotin treatment	Incubation time
BirA*	37 kDa	*O. sativa* (Lin et al., 2017)	NA	Leaf protoplasts	Submerged in 50 µM biotin in WI solution	24 h
		*N. benthamiana* (Conlan et al. 2018)	25 °C	Leaf	Infiltration of 75 µM biotin solution	24 h
		*A. thaliana*	22 °C, 25 °C	Leaf (Khan et al. 2018)	Infiltration with 2 mM biotin solution	24 h
				Seedlings (Miltenburg et al., 2022)	Submerged in 1 mM biotin solution	24 h
BioID2	27 kDa	*A. thaliana* (Huang et al., 2020; Tang et al. 2020; Tang et al. 2022b; Jia et al. 2023; Tang et al., 2024; Kenneally et al., 2026; Nam et al., 2026)	22 °C, 25 °C	Seedlings	Submerged in 50 μM biotin solution	16 h, 24 h
TurboID	35 kDa	*A. thaliana*	22 °C, 25 °C	Seedlings (Mair et al. 2019; Xu et al., 2021; Jia et al. 2023; Kim et al., 2023; Fang et al., 2025; Nam et al., 2026)	Submerged in a 50 µM biotin solution	3 h, 4 h, 5 h, 6 h
				Leaf (Xu et al., 2021)	Infiltration with 50 μM of biotin solution	6 h
				Inflorescence stems with young flower buds (Feng et al., 2023)	Submerged in 0.5 mM biotin solution and incubated under light	6 h
		*N. benthamiana* (Zhang et al., 2019; Kim et al., 2023; Zhang et al., 2023; Hamada et al., 2026)	25 °C	Leaf	Infiltration with 50 to 200 µM biotin in 10 mM MgCl_2_ solution	3 h, 8 h, 12 h
		*C. reinhardtii* (Kreis et al., 2023; Lau et al. 2023)	22 °C	Culture	Resuspended in media supplemented with 1 to 2.5 mM biotin solution	4 h
		*O. sativa* (Shi et al., 2024)	25 °C	Seedling	Submerged in 50 μM biotin after 24 h starvation in distilled water	12 h
		*Fragaria × ananassa* (Martín-Pizarro et al., 2026)	NA	Fruit	Ripe strawberry fruits have high biotin levels naturally (∼15 ng/g fresh weight)	NA
		*Phaeodactylum tricornutum* (Zhang et al., 2025b)	20 °C	*biob* (biotin synthase) mutant	Incubated in culture media supplemented with 100 μM biotin	3 h
Split-TurboID	Split site: G78/G79 N-ter: 8 kDa C-ter: 27 kDa (Huang et al., 2025)	*A. thaliana*	25 °C	Seedling	Submerged in 50 µM biotin solution	30 min
	Split site: R141/G142 N-ter: 15 kDa C-ter: 20 kDa (Zhang et al., 2024)	*N. benthamiana*	25 °C	Leaf	Infiltration with 200 µM biotin in 10 mM MgCl_2_ solution	8 h
dCas9–TurboID	dCas9: 100 kDa (dependent on the dCas9 species) TurboID: 35 kDa	*A. thaliana* (Zhang et al., 2026)	NA	Leaf	Sprayed with a 100 μM biotin solution containing 0.005% Silwet L-77	Once every hour for a total treatment time of 3 hours
		*N. benthamiana* (Zhang et al., 2025a)	NA	Leaf	Infiltration with 200 µM biotin in infiltration solution	12 h
		*B. oleracea* (Zhang et al., 2026)	NA	Leaf	Immersed 3 times in 200 μM biotin solution after dewaxing	10 min each time, at one-hour intervals
		*O. sativa* (Zhang et al., 2026)	NA	Leaf	Immersed 3 times in 200 μM biotin solution	10 min each time, at one-hour intervals
miniTurbo	28 kDa	*A. thaliana* (Tang et al., 2024; Olson et al., 2025)	25 °C	Seedlings	Submerged in a 50 µM biotin solution	6 h
AirID	37 kDa	*C. sativus* (Zada et al. 2022)	23 °C	Leaf	Infiltration of 5 µM biotin in 10 mM MgCl_2_ solution	8 h
UltraID	20 kDa	Not used in plants yet				
BASU	29 kDa	Not used in plants yet				

For applications requiring minimal steric interference, such as probing membrane-associated complexes or structurally constrained interaction interfaces, smaller enzymes with moderate activity (eg, miniTurbo or BioID2) are preferable, as they reduce the risk of tag-induced disruption and limit nonspecific background. For capturing transient or stimulus-responsive interactions, fast-labeling enzymes (eg, TurboID) are better suited given their rapid kinetics and compatibility with short labeling windows. It is also worth noting that no single enzyme is optimal across all contexts: TurboID offers the highest labeling efficiency but can produce elevated background, whereas miniTurbo trades some signal intensity for improved specificity. For specialized applications such as split-enzyme systems or CRISPR-based PL, enzyme selection additionally influences reconstitution efficiency and signal-to-noise ratio and should be evaluated empirically. Moreover, due to the wide variation in activity and kinetics among biotin ligases, applying a new enzyme in plants requires careful optimization to balance robust target biotinylation against nonspecific background labeling and signal saturation.

### Selecting expression systems and promoters

The choice of expression system, promoter, and tissue context substantially shapes PL outcomes and should be aligned with the biological question under investigation. Transient expression systems, such as agroinfiltration in *N. benthamiana* or protoplast transfection, offer speed and experimental flexibility, and are well suited for initial validation of bait-fusion functionality and labeling efficiency. However, transient and heterologous expression systems typically drive high bait accumulation in a non-native protein environment, which can introduce artifacts, particularly when the bait protein lacks spatial constraint. Inducible expression systems mitigate some of these concerns by providing temporal control and reducing the risk of chronic overexpression artifacts, though they still require careful calibration of induction levels.

Stable transgenic lines provide the most physiologically relevant context for PL experiments and are strongly preferred for publication-quality datasets. When generating stable lines, native promoters should be used wherever feasible to preserve endogenous expression levels and spatiotemporal patterns. If native promoter-driven expression proves insufficient (typically evidenced by weak immunoblot signal and a poor prey signal-to-background ratio), constitutive promoters such as Cauliflower Mosaic Virus 35S or *Ubiquitin* (UBQ) promoters may be used, with the caveat that overexpression artifacts should be assessed. If constitutive promoters are used, lines with “moderate” bait expression are preferable to high-expressing lines, as they reduce nonspecific background and improve discrimination of biologically meaningful proximity partners. Note that “moderate expression” describes bait expression levels that generate a readily detectable, but not excessive, bait immunoblot signal while maintaining a clear prey signal-to-background ratio. With the advent of cell-type-resolved plant transcriptomic atlases, the use of non-native yet cell type- or tissue-specific promoters to achieve an appropriate expression pattern of bait proteins has become increasingly feasible and is encouraged when functional specificity is a priority.

### Designing functional fusion proteins

Fusion protein design is another critical determinant of PL data quality, as mislocalized or nonfunctional bait constructs will produce misleading proximity datasets regardless of experimental rigor downstream. Prior to committing to large-scale PL experiments, the functionality and correct localization of the fusion construct should be validated. At a minimum, fluorescent protein fusions should be generated to assess whether N- or C-terminal tagging preserves the native subcellular localization and, when feasible, the biological function of the bait protein. Alternatively, immunofluorescence imaging of the biotin ligase fusion proteins can be performed. Tandem fusions combining a fluorescent protein with a biotin ligase enable simultaneous visualization and labeling from a single construct, though the substantially increased tag size warrants additional functional validation. The gold standard for functional validation is complementation of a loss-of-function mutant using biotin ligase-fused bait constructs.

For transmembrane proteins, determining membrane topology before construct design is essential, as the biotin ligase must be positioned on the membrane face relevant to the biological question. In addition, for certain transmembrane proteins, N-terminal fusion of TurboID can result in extensive nonspecific labeling of proteins involved in cotranslational translocation, including signal recognition particles (SRP), SRP receptors, and ribosomes. In some cases, neither terminus is accessible or appropriate, necessitating internal tag insertion (Nam et al. 2026); such designs require particular care to avoid disrupting functional domains. Regardless of insertion strategy, flexible linkers between the bait protein and the biotin ligase are recommended, as they accommodate conformational freedom, extend the effective labeling radius, and reduce steric hindrance that might otherwise compromise both enzymatic activity and bait protein function (Kim et al. 2016). For bait proteins involved in protein turnover, such as E3 ligase adaptors or proteasome-associated factors, validation should consider whether the PL fusion perturbs the native degradation pathway itself, for instance, by altering substrate stability, complex stoichiometry, or stress-responsive signaling outputs (Sun et al. 2024; Li et al. 2025).

Advanced PL platforms often require additional system-specific validation beyond confirming bait functionality and localization. For split-enzyme approaches such as split-TurboID, it is important to verify that biotinylation activity is reconstituted only when the 2 enzyme fragments are brought together by the intended biological interaction or condition, using appropriate controls to assess background labeling. Likewise, CRISPR-based PL approaches require validation of accurate genomic targeting and locus specificity, which can be confirmed using orthogonal methods such as ChIP-seq or CUT&RUN to ensure that the labeling enzyme is recruited to the intended genomic region.

## Performing the proximity labeling proteomics

### Biotin delivery and labeling window across plant tissues and species

Plant tissues present distinctive physical barriers to biotin delivery, including rigid cell walls, extracellular polysaccharide matrices, and intercellular air spaces that restrict substrate diffusion and may retain biotin after entry. In Arabidopsis, cellular biotin uptake occurs at least in part through transporter-mediated mechanisms, including SUCROSE TRANSPORTER 5 at the plasma membrane (Pommerrenig et al. 2013), although passive diffusion driven by high extracellular concentrations may also contribute (Bowman et al. 1986). In practice, submerging Arabidopsis tissues, such as seedlings or detached leaves, in biotin-supplemented liquid media (typically 10 to 50 µM) is sufficient to achieve efficient proximity-dependent biotinylation in many experimental contexts.

For structurally complex or less permeable tissues, mechanical delivery methods such as syringe or vacuum infiltration can overcome diffusion barriers and improve substrate penetration. Biotin accessibility should be empirically assessed for each tissue type, as penetration can vary substantially even within a single species, for example, between intact floral buds and seedlings in Arabidopsis (Mair et al. 2019). Evidence from genetic studies further suggests that biotin uptake and long-distance transport may influence labeling efficiency in whole-plant experiments. Patton et al. (1998) demonstrated that vegetative growth of the Arabidopsis *bio2* mutant, which is defective in biotin biosynthesis, can be rescued by supplying exogenous biotin through the growth medium or irrigation water. However, these plants failed to bolt after transfer to soil despite continued watering with 1 mM biotin, suggesting that biotin absorbed from the root may not be transported efficiently to all aboveground tissues or the amount of biotin absorbed may be insufficient to meet the demands of reproductive development or protein labeling. Therefore, root application alone may not achieve consistent labeling across all tissues.

**Figure 1:**
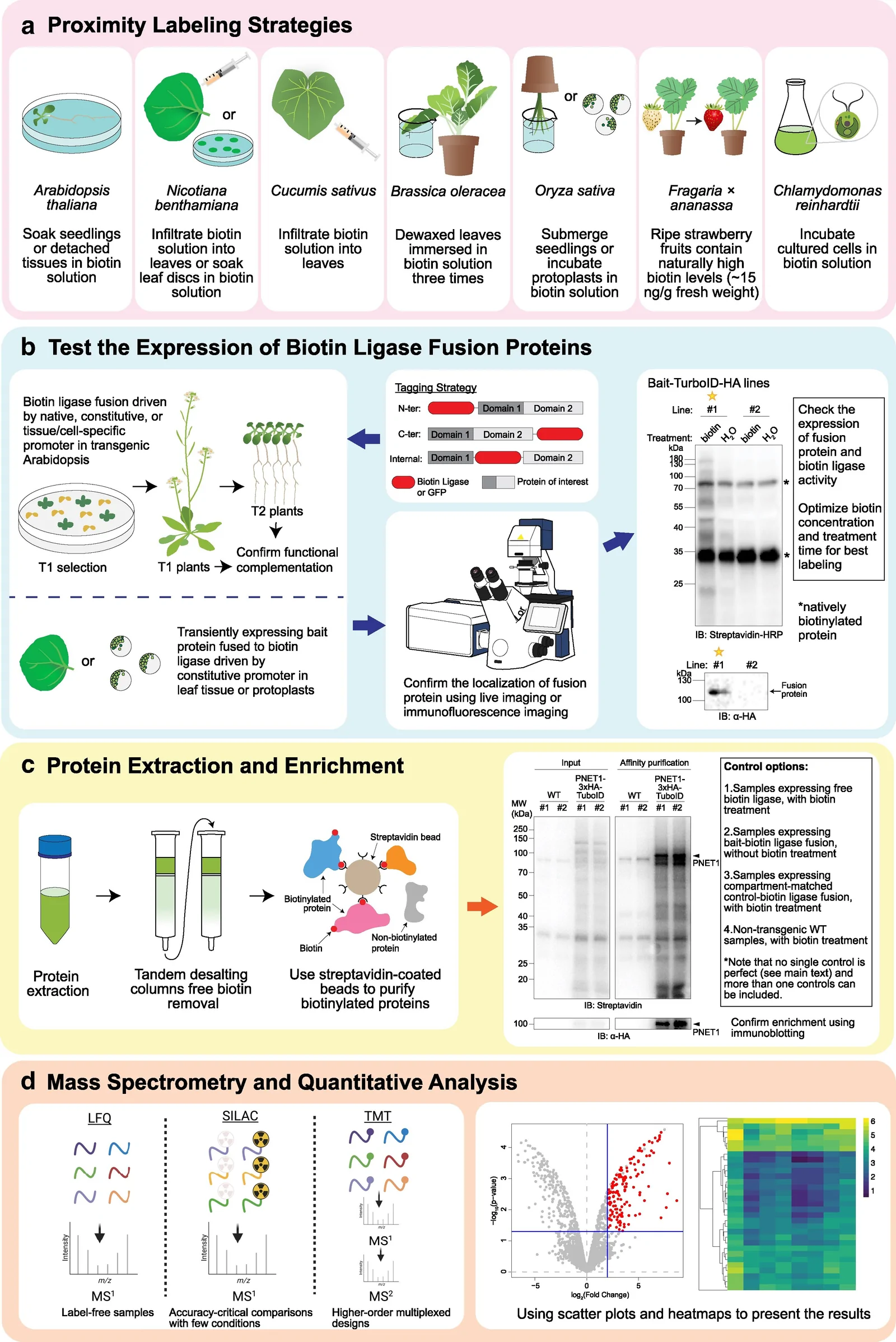
Workflow for proximity labeling optimization and analysis in plants. A, Species-specific biotin delivery. Effective PL requires tailored biotin concentrations and application methods. B, Design of fusion proteins and validation of their expression and activity. Before scaling, bait-biotin ligase fusions must be verified in transgenic (T1/T2) or transient systems for correct localization. After confirming protein localization, Western blot is performed to identify successful lines (indicated by yellow stars) with robust streptavidin-HRP signals following biotin treatment compared to controls. C, Enrichment of biotinylated proteomes. Following protein extraction, tandem desalting columns are used to remove free biotin, which otherwise competes for binding of biotinylated proteins during affinity purification. Selective capture of the proxiome is achieved using streptavidin-based affinity purification (Tang and Gu, 2025). Western blots confirm the significant enrichment of biotinylated candidates (eg, PNET1) in the purified fraction relative to the input. Control options are listed in the box. Immunoblot images are adapted from Fang et al. 2025. D, Quantitative proteomics and data visualization. Enriched samples are processed via LFQ, SILAC, or TMT pipelines (Mair et al. 2019; Zuniga et al. 2024). High-confidence candidates are identified through statistical analysis and visualized using scatter/volcano plots to show fold-change and statistical significance and heatmaps to demonstrate experimental reproducibility and clustering.

Different plant species require tailored biotin treatment strategies reflecting their distinct anatomical and physiological characteristics (Fig. 1A and Table 1). In *C. reinhardtii*, labeling conditions parallel those used for Arabidopsis seedlings but require substantially higher biotin concentrations (1 to 2.5 mM); a 4-hour TurboID incubation is sufficient for efficient labeling, and this system has been productively applied to investigate thylakoid biogenesis, stress responses, and the pyrenoid proxiome (Kreis et al. 2023; Lau et al. 2023). In *N. benthamiana*, transiently expressing tissues can be labeled by incubating leaf discs in 250 µM biotin solution for 1 h, or by syringe-infiltrating 50 or 200 µM biotin in a 10 mM MgCl_2_ solution 36 to 60 h post-agroinfiltration and harvesting 3 to 12 h later (Mair et al. 2019; Zhang et al. 2019, 2023; Hamada et al. 2026). Adding MgCl_2_ during leaf infiltration maintains cellular homeostasis and prevents ion efflux, which may ultimately improve biotin absorption. In crop species, where generating stable transformants is resource-intensive, transient expression in leaves or protoplasts offers a practical alternative: *Cucumis sativus* leaves agroinfiltrated with AirID fusion constructs can be labeled with 5 µM biotin at 36 h post-transformation and harvested 4 to 12 h later (Zada et al. 2022), while rice protoplasts have been successfully labeled using BirA* with 50 µM biotin over 24 h (Lin et al. 2017). In stable transgenic rice lines, ten-day-old rice seedlings were first starved via incubation in distilled water for 24 h and then submerged in 50 μM biotin solution for 12 h at room temperature for labeling (Shi et al. 2024). In addition, species-specific tissue properties must be accommodated: *B. oleracea* leaves require dewaxing prior to 3 repeated immersions in 200 µM biotin (10 min each, at 1-hour intervals) (Zhang et al. 2026). Proximity labeling has recently been utilized in *Fragaria*  *×*  *ananassa* (strawberry) to study fruit ripening. In this approach, Agrobacterium expressing TurboID fused to a gene of interest was infiltrated into white-stage fruits, followed by exogenous biotin supplementation at the red-fruit stage. Notably, because ripe strawberry fruits naturally contain high endogenous biotin levels (∼15 ng/g fresh weight), Martín-Pizarro et al. (2026) observed no significant differences in global protein biotinylation between fruits with or without additional biotin supplementation.

The literature values summarized in Table 1 should serve as initial guidelines rather than universal rules. Because proximity labeling depends on both enzyme kinetics and plant physiology, key parameters, including biotin concentration, incubation time, temperature, and delivery method, must be optimized for each system. When working with a new species, tissue, or developmental stage, conduct pilot experiments to maximize target signal while minimizing background noise and physiological disruption. Beyond technical optimization, the specific duration of the labeling window also dictates the type of biological insights captured. Longer labeling periods increase recovery of weak or transient proximity partners but integrate cumulative interaction history, while shorter labeling windows provide greater temporal resolution and are better suited for rapid signaling events, although they may reduce prey recovery and require stronger bait expression or more sensitive MS acquisition.

### Selecting transgenic lines

Once biotin delivery conditions have been established, appropriate transgenic lines must be identified (Fig. 1B). Biotin ligase fusions are typically tagged with a small epitope (eg, HA, Myc) to enable immunoblot-based detection. Multiple independent T1 lines should be screened for fusion protein expression. T1 or T2 plants should then be subjected to biotin treatment alongside mock-treated controls, with protein extracts probed by streptavidin-HRP immunoblotting to confirm active biotinylation. This validation step is essential, as not all lines that express the fusion protein exhibit detectable biotinylation activity, the basis for which remains poorly understood. The bait protein itself is frequently self-biotinylated, providing a convenient internal indicator of ligase activity. Lines that express the fusion protein at the correct size but show little or no biotinylation signal likely suffer from low expression, protein instability, or misfolding, and should be deprioritized. Lines exhibiting moderate, stable bait expression with clear biotin-inducible labeling compared to mock controls are optimal for downstream proteomic analysis, as they balance labeling efficiency with reduced nonspecific background. Excessively high bait expression can expand the effective labeling radius, increase background, and distort native stoichiometry. Therefore, moderate expression lines that retain expected localization and function are generally preferable to maximal expressers.

### Protein extraction and enrichment

Efficient recovery of biotinylated proteins requires extraction conditions stringent enough to solubilize membrane-associated proteins while preserving the biotin–streptavidin interaction. Mild nonionic detergents, such as 0.5% Triton X-100, 0.5 to 1% sodium deoxycholate, or 0.5 to 1% Nonidet P-40, are compatible with streptavidin-based enrichment and are widely used for this purpose (Tang et al. 2020; Li et al., 2025). Reducing agents must be strictly avoided prior to streptavidin pulldown, as they disrupt the biotin–streptavidin interaction and markedly impair enrichment efficiency. For bait proteins with well-defined subcellular localization (eg, specific organelles), subcellular fractionation can be performed prior to protein extraction to enrich the target compartment and reduce background from unrelated cellular components.

Prior to affinity purification, protein extracts must be thoroughly desalted to remove excess free biotin, which competes with biotinylated proteins for streptavidin binding; simple rinsing is insufficient, and tandem desalting columns or FPLC-based buffer exchange are required (Fig. 1C). Importantly, with highly active ligases such as TurboID, residual enzymatic activity may persist transiently after tissue disruption if free biotin remains present. Because cell lysis abolishes native compartmentalization and greatly increases protein diffusion, continued post-lysis labeling can generate ex vivo nonspecific biotinylation events that do not reflect the in vivo proximity environment. To minimize this risk, extraction should be performed rapidly and at cold temperature, with prompt clarification of cell lysate and immediate removal of free biotin before prolonged handling steps. Additionally, although protease inhibitors reduce proteolysis in cell lysates, prolonged incubation with affinity beads can still reduce yield due to the gradual degradation of both bait and target proteins. Following pulldown, sequential washes of increasing stringency are recommended to minimize nonspecific protein binding to beads.

Before proceeding to mass spectrometry, quality control immunoblotting (eg, anti-HA antibody) of both input and pulldown fractions should be performed to confirm fusion protein expression, solubility, and recovery, while streptavidin-based detection (streptavidin-HRP) should demonstrate biotin-dependent protein labeling and enrichment relative to nontransgenic or biotin-mock treatment controls. A weak signal of the bait protein or its inducible biotinylation relative to mock-treated controls typically indicates inefficient labeling, extraction, or affinity purification, and often predicts low-quality MS data with elevated false-positive rates and should prompt re-optimization before proceeding.

### Mass spectrometry and data analysis

For label-free quantitative (LFQ) proteomics, a minimum of 2 to 3 biological replicates per condition is recommended, including both biotin-treated samples and appropriate negative controls. Baseline control conditions, including ligase-only controls (biotin ligase expressed without a bait fusion), bait-fusion lines without exogenous biotin treatment, and compartment-matched controls (biotin ligase fused to control protein localized to the same subcellular compartment as the bait), serve distinct purposes in assessing bait-dependent enrichment, background biotinylation, and spatial specificity, respectively. However, no single control is universally optimal, and each carries inherent limitations. For example, controls expressing free biotin ligase or biotin ligase fused to a freely diffusing protein such as GFP often generate false positives, as their high abundance and lack of spatial restriction may lead to widespread background labeling that masks genuine bait-specific enrichment. Similarly, transgenic lines expressing the bait fusion without exogenous biotin treatment may also yield false negatives, although to a lesser extent, because endogenous biotin can support low levels of biotinylation, resulting in labeling of bona fide proximal proteins even in the absence of biotin supplementation and consequently reducing apparent enrichment. Therefore, control selection should be guided by the biological question, the subcellular localization of the bait, and the specific PL system employed. In practice, nontransgenic plants treated with biotin serve as effective controls for excluding most endogenously biotinylated plant proteins.

Differential enrichment analysis between experimental and control groups can be performed using established pipelines such as the DEP (Differential Enrichment analysis of Proteomics data) package in R (Zhang et al. 2018; Tang and Gu, 2025), with results visualized as volcano plots, heatmaps, or interaction network diagrams. Enriched proteins are commonly selected either by using known interactors to establish appropriate significance thresholds or by applying empirical criteria, such as a fold-change greater than 2 (compared to control) and a *P* value below 0.05. The resulting candidate list is typically further refined by applying a peptide spectrum match (PSM) threshold (eg, PSM > 1) and by cross-validating enrichment using spectral count data, requiring higher PSM values in experimental samples than in corresponding controls. Integration with PPI databases and co-expression datasets can further support biological interpretation and help prioritize candidates for downstream validation. Before performing such analyses, however, common contaminants and recurrent artifacts, including highly abundant cellular proteins such as ribosomal proteins, should be carefully evaluated and, where appropriate, removed from the dataset. This consideration is particularly important for certain bait proteins (eg, transmembrane protein) fused to an N-terminal biotin ligase, as the ligase may become active during cotranslational synthesis, enabling biotinylation of ribosomes and other components of the translation machinery before the full-length bait protein has been synthesized and localized to its native cellular compartment.

It bears emphasis that PL datasets define proximity-based proteomes, not direct interaction networks; proximity enrichment should therefore not be equated with physical binding without corroborating biochemical evidence. Indeed, because PL enriches proteins within the spatial labeling radius of a bait rather than distinguishing direct binding from indirect proximity, terms such as proxiome, proxitome, or proximitome are preferable for PL-enriched protein sets, whereas interactome should be reserved for associations supported by orthogonal validation.

Beyond LFQ approaches, several metabolic and chemical labeling strategies are available to quantify proteomic differences between samples and controls in plant research (Fig. 1D). SILAC (Stable Isotope Labeling by Amino acids in Cell culture) and its plant-adapted counterpart SILIA/SILIP (Stable Isotope Labeling in Arabidopsis/Planta) rely on metabolic incorporation of heavy isotopes to achieve high quantitative accuracy, although they are typically limited to 2 to 3 multiplexed samples (Shrestha et al. 2022). In contrast, Tandem Mass Tag (TMT) labeling enables simultaneous analysis of up to 35 samples through isobaric chemical tagging (Zuniga et al. 2024). These approaches allow labeled proteins (SILAC and SILIA) or peptides (TMT) to be pooled early in the workflow, enabling the combined sample to undergo extensive fractionation. This reduces sample complexity, enhances detection sensitivity, and broadens the dynamic range of the proteomic measurement. Overall, metabolic labeling methods (SILAC/SILIA/SILIP) are best suited for accuracy-critical comparisons with few conditions, leveraging early pooling for deep, high-confidence quantification. Conversely, TMT-based approaches are ideal for higher-order multiplexed designs that simultaneously evaluate multiple genotypes, treatments, or time points.

## Quantitative and conditional proximity labeling proteomics

Increasingly, plant proximity labeling experiments are moving beyond static cataloging of bait-proximal proteins toward quantitative comparisons across treatments, genotypes, time points, and physiological states. In these cases, PL should be designed as a comparative proteomics experiment from the outset. This requires matched controls, sufficient biological replications, consistent biotin delivery, randomized sampling, and quantitative MS strategies that can distinguish true changes in proximity from differences in bait abundance, tissue physiology, labeling efficiency, or total protein abundance.

TMT-based workflows are particularly useful for quantitative differential analysis to compare proxiome under various conditions, as samples can be multiplexed, pooled early, fractionated together, and analyzed with reduced technical variation (Mao et al. 2021; Zuniga et al. 2024; Reyes et al. 2026). This approach is especially valuable for plant stress or hormone-response experiments, where biological variability among plants, treatments, or infection states can be substantial. For example, TurboID-based profiling of ASK1/SCF-associated networks in Arabidopsis has been combined with TMT proteomics to compare basal, drought-stressed, and pathogen-infected conditions, revealing condition-specific remodeling of F-box proteins, proteasome-associated factors, transcriptional regulators, and hormone-related proteins (Sun et al. 2024; Rodriguez-Zaccaro et al. 2025; Hamada et al. 2026). These studies illustrate how quantitative PL can resolve dynamic protein neighborhoods that are not apparent from single-condition datasets.

In practice, robust TMT-based PL workflows require attention to labeling efficiency, balanced peptide loading across channels, and appropriate downstream statistical analysis. Database searches should account for fixed carbamidomethylation of cysteine, common variable modifications such as methionine oxidation and protein N-terminal acetylation, and TMT labeling on peptide N-termini and lysine residues. False discovery rates of 1% at both peptide spectrum match and protein levels are typically applied, and stringent quantitative filtering is recommended to minimize ratio distortion arising. While TMT provides robust multiplexed quantification, advances in LFQ methods, particularly data-independent acquisition (DIA), can offer greater proteome depth at lower costs, with some studies reporting improved identification of low-abundance proteins compared to TMT-based workflows (Zhong et al. 2024; Jang et al. 2026), though TMT multiplexing retains practical advantages for experimental designs comparing multiple conditions simultaneously.

Conditional PL datasets also require cautious interpretation. A protein enriched under one condition may reflect increased physical proximity to the bait, altered subcellular localization, changes in total prey abundance, changes in bait abundance, or differences in labeling accessibility. Moreover, TMT-based workflows are susceptible to ratio compression, in which co-isolated peptide ions dampen measured fold-change values and may obscure genuine low-abundance interactors (Hogrebe et al. 2018). Whenever possible, PL proteomics datasets should be interpreted alongside supporting measurements, such as input immunoblots, bait expression levels, whole-proteome data, or orthogonal validation assays. For stress- or hormone-regulated systems, whole-proteome measurements can be particularly informative because they help distinguish remodeling of the proxiomes from global protein abundance shifts. Critically, the use of stably transformed isogenic lines ensures that differential enrichment between conditions reflects genuine stress-responsive network remodeling rather than artifacts of control bait expression context or targeting efficiency.

Finally, quantitative PL is best viewed as a prioritization framework for identifying condition-responsive candidate proteins rather than definitive evidence of direct interaction. Candidates emerging from comparative datasets should be followed by validation approaches appropriate to the biological question, such as co-immunoprecipitation, reciprocal PL, BiFC/split-luciferase assays, genetic analysis, degradation assays, or biochemical reconstitution.

## Future directions

The rapid expansion of PL technologies is transforming how molecular networks can be interrogated in plant systems. Continued engineering of PL enzymes to improve catalytic efficiency at plant-compatible temperatures, reduce basal background activity, and enhance temporal precision will further increase their compatibility with plant physiology. Such improvements will be particularly important for studying rapid signaling events, dynamic membrane contact sites, and environmentally responsive protein assemblies that form and dissolve over short timescales.

Looking ahead, the field would benefit from continued diversification of labeling chemistries and substrate systems. Developing next-generation ligases with improved specificity and tunable kinetics, or engineering alternative substrate-based PL strategies that avoid oxidative stress or endogenous biotin competition, could further enhance accuracy and flexibility. The ability to deploy multiple orthogonal labeling systems in parallel may ultimately enable multiplexed proximity profiling, tracking distinct protein populations or dynamic relocalization events within the same cell. Integrating cross-linking mass spectrometry could further refine the mapping of protein–protein interaction networks and subcellular proteomes. Additionally, incorporating stimuli or treatments to resolve “conditional proxiomes” represents a high-priority direction for future work. Ultimately, the greatest impact may come from integrating proximity labeling with quantitative whole-proteome, phosphoproteome, ubiquitylome, and other multi-omic datasets to build predictive models that connect dynamic protein neighborhoods with signaling pathways, protein turnover, and cellular function across developmental and environmental contexts.

## Data Availability

No new data were generated or analysed in support of this research.
